# Use of ß-blockers and mortality following ovarian cancer diagnosis: a population-based cohort study

**DOI:** 10.1186/1471-2407-13-85

**Published:** 2013-02-22

**Authors:** Sigrun A Johannesdottir, Morten Schmidt, Gary Phillips, Ronald Glaser, Eric V Yang, Michael Blumenfeld, Stanley Lemeshow

**Affiliations:** 1 Division of Biostatistics, College of Public Health, The Ohio State University, Columbus, OH, USA; 2 Department of Clinical Epidemiology, Aarhus University Hospital, Aarhus N, Denmark; 3 The Ohio State University, Center for Biostatistics, Columbus, OH, USA; 4 The Ohio State University Medical Center, Columbus, OH, USA; 5 Institute for Behavioral Medicine Research, The Ohio State University, Columbus, OH, USA; 6 Comprehensive Cancer Center, The Ohio State University Medical Center, Columbus, OH, USA

## Abstract

**Background:**

Experimental data suggest that catecholamine hormones are involved in stimulating the aggressiveness of ovarian cancer, but few population-based studies have examined this association. We therefore conducted a population-based cohort study to examine whether ß-blockers affect mortality following ovarian cancer diagnosis.

**Methods:**

We used the Danish Cancer Registry to identify all patients diagnosed with ovarian cancer in northern Denmark between 1999 and 2010 (n=6,626). Data on medication use, comorbidity, and survival were obtained from medical databases. According to the last redeemed prescription before diagnosis, ß-blocker use was categorized as current (within ≤90 days), previous (>90 days) or never. We used Cox proportional hazards regression to calculate hazard ratios (HRs) for all-cause mortality with 95% confidence intervals (CIs) adjusting for confounding factors.

**Results:**

Among the ovarian cancer patients, 373 (5.6%) were current, 87 (1.3%) previous, and 6,166 (93.1%) were nonusers of ß-blockers. Median duration of use was 19.0 months among current users and 43.0 months among previous users. Median follow-up was 2.55 years (IQR: 0.81-9.23). Nonusers and current users of ß-blockers had similar comorbidity burden whereas previous users had moderate comorbidity more frequently. Compared with nonusers, the adjusted HR was 1.17 (95% CI: 1.02–1.34) for current users and 1.18 (95% CI: 0.90–1.55) for previous users. Secondary analyses stratifying by cancer stage and duration of ß-blocker use supported the overall results.

**Conclusions:**

We found no evidence that ß-blocker use was associated with decreased mortality following ovarian cancer diagnosis.

## Background

 Inhibiting the sympathetic actions of catecholamine hormones (*i*.*e*., epinephrine and norepinephrine), ß-blockers are used for various indications, particularly cardiac arrhythmias, cardioprotection after myocardial infarction, hypertension, migraine, and tremor [1]. These diverse indications reflect the abundance of ß-adrenoceptors in the body. Experimental evidence shows that malignant cell lines from, *e*.*g*., ovarian cancer and malignant melanoma also express ß-adrenoceptors and that catecholamine stress hormones may affect carcinogenesis through these receptors [2-8].

 Previous research on the association between ß-blocker use and mortality following malignant melanoma, have shown consistent results between the protective effects observed ex-vivo and in a population-based setting [7-9]. However, data on the effect of ß-blockers on mortality following ovarian cancer in a population-based setting are sparse [10]. We therefore conducted a population-based cohort study to examine whether use of ß-blockers are associated with mortality in patients with ovarian cancer.

## Methods

### Setting

 The Danish National Health Service guarantees the entire Danish population universal tax-supported health care including access to general practitioners and hospitals as well as partial reimbursement of various drugs including ß-blockers [11-13]. All individuals residing in Denmark at any point in time since 1968 are assigned a unique central personal registration (CPR) number, which is used to record health-related services in various nationwide registries and allows accurate and unambiguous individual-level linkage of all registries [13].

 We conducted this population-based cohort study in northern Denmark (population 1.7 million, ≈30% of the Danish population). This region encompasses the former North Jutland County, Aarhus County, Viborg County and Ringkøbing County for which complete computerized prescription records are available through the Aarhus University Prescription Database since 1 January 1998 [12]. By starting the study period on 1 January 1999, we ensured a minimum of one year of prescription history for all participants in the study.

### Study cohort

The Danish Cancer Registry (DCR) has recorded information on all incident malignant neoplasms in Denmark since 1943 [14]. Tumors are classified according to the 7^th^ revision of the International Classification of Diseases (ICD-7) from 1943 through 2003 and according to the 10^th^ revision thereafter [14].

 We used the DCR to identify all women with a first-time diagnosis of ovarian cancer from 1 January 1999 to 31 December 2010. We also included information on stage at diagnosis according to the Summary Staging classification with the TNM grouping translated as follows: localized (TNM: T1–4, N0, M0), regional (TNM: Tx, N1–3, M0), distant (TNM: Tx, N1–3, M1), or unknown/missing. We included only women aged 20 years or more at time of diagnosis.

### ß-blocker use

 Using the Aarhus University Prescription Database [12], we identified all prescriptions for ß-blockers redeemed by study subjects before their diagnosis date. For each prescription dispensed, the patient’s CPR number, type and amount of drug prescribed according to the Anatomical Therapeutic Chemical (ATC) classification system, and date of dispensation, are recorded in the electronic accounting system at the pharmacy and subsequently transferred to the database [12].

 We defined three exposure categories: (1) current users were those redeeming at least one prescription within 90 days of ovarian cancer diagnosis, (2) previous users were those redeeming their last prescription in the interval 91 to 365 days prior to ovarian cancer diagnosis, and (3) nonusers were those with no prescription records of ß-blocker use within 365 days of ovarian cancer diagnosis. We assessed exposure status prior to diagnosis to avoid introducing immortal time bias, *i*.*e*., that users would appear to survive longer because a user by definition had to survive to become a user [15]. We chose to assess exposure prior to diagnosis to mimic an intention-to-treat method [9] and because we hypothesized that ß-blockers exert an inhibitory effect early in ovarian cancer progression. In Denmark, most prescriptions cover 30 to 90 days. To assure that we captured most current users, we therefore chose 90 days as the exposure window.

### Other patient characterstics

 We used the Aarhus University Prescription Database to obtain information on comedication use of the following agents between establishment of computerized prescription registries in each county and the date of ovarian cancer diagnosis: angiotensin-converting enzyme (ACE) inhibitors, calcium channel blockers, diuretics, statins, antidepressant drugs, antipsychotics, anxiolytic drugs, and hormone replacement therapy. Over-the-counter use of aspirin and low-dose ibuprofen is not recorded in the database [12]. However, regular users are typically registered in the prescription database because the cost is automatically partly refunded when a physician prescribes the drug [12].

 The Danish National Registry of Patients (DNRP) has recorded all inpatient admissions to non-psychiatric hospitals since 1977, and all outpatient and emergency admissions since 1995 [16]. Each record includes information on dates of admission and discharge, diagnosis codes and associated surgical procedures [16]. Diagnoses are classified according to ICD-8 through 1993 and the ICD-10 revision thereafter [16]. Surgical procedures are recorded using a Danish version of the Nordic Medico-Statistical Committee (NOMESCO) Classification of Surgical Procedures [16]. We used the DNPR to retrieve information on any diagnosis of obesity, atrial fibrillation/flutter, angina pectoris, myocardial infarction, congestive heart failure, esophageal varices, tremor, anxiety, thyrotoxicosis, chronic obstructive pulmonary disease (COPD), migraines, stroke, chronic kidney disease, or a history of hysterectomy or tubal sterilization prior to ovarian cancer diagnosis. We also measured the comorbidity burden using the Charlson Comorbidity Index (CCI) categorized as low (score 0), moderate (score 1–2), or high (score ≥3). The CCI is an extensively studied and validated instrument used to predict the risk of death from comorbid diseases, by covering and weighing 19 major chronic disease categories based on the relative risk of dying [17-21].

All ICD and ATC codes used in the study are provided in the Additional file 1: ATC and ICD codes.

### Mortality

We started follow-up on the date of diagnosis and continued until death, emigration, or end of follow-up (31 December 2010) whichever came first. We identified all-cause mortality using the Danish Civil Registration System. The Danish Civil Registration System was established on 1 April 1968 and includes daily updates on changes in migration and vital status for the entire Danish population [13].

### Statistical analysis

Initially, we computed the frequency and proportion of covariates, number of deaths, and amount of accumulated person-time within categories of ß-blocker use. Using time since diagnosis as the time scale, we then used Cox proportional hazard regression to estimate hazard ratios (HRs) with 95% confidence intervals (CIs) associating ß-blocker use with mortality both overall and according to stage at diagnosis. We used a risk-factor modeling approach to fit a multivariable model. Age group (20–40, 41–60, 61–80, and >80 years) and CCI level were variables of interest a priori and therefore included in the model from the start. Adding the remaining variables to the model one at the time resulted in a less than 10% change in the HR associated with ß-blocker use. However, including prior use of diuretics, aspirin, and statins, collectively in the model demonstrated substantial confounding (>10%) and they were therefore included in the model. We then tested for statistically significant interactions between any of the covariates and ß-blocker use. We assessed the assumption of proportional hazards by graphical examination of log-log plots and found it not to be violated.

 We performed two secondary analyses according to duration of use (calculated as time between first and last prescription plus 90 days). First, we made a restriction to current users with at least one year of ß-blocker use and compared them to non-users. Second, we examined the association between ß-blockers and mortality following ovarian cancer according to months of use among all user groups. To ensure at least five years of prescription history and thereby limit left censoring, this analysis was restricted to women diagnosed in 2003–2010. The method of fractional polynomials was used to confirm that months of use, was linear in the log-hazard function.

 We performed all analyses using STATA® software (version 11.0, STATA, College Station, TX). The study was approved by the Danish Data Surveillance Authority.

## Results

### Patients characteristics

Table 1 presents the characteristics of the 6,626 ovarian cancer patients included in the study. Median age at diagnosis was overall 65 years (between 63–66 years for all exposure groups). There were 6,166 (93.1%) nonusers, 373 (5.6%) current users, and 87 (1.3%) previous users of ß-blockers in the cohort (Table 1). The median duration of use was 19.0 months among current users and 43.0 months among previous users (Table 1).

**Table 1:** **Ovarian cancer patient demographics by ß**-**blocker use**

	**Nonusers**	**Current users**	**Previous users**
Number (%)	6,166 (93.06)	373 (5.63)	87 (1.31)
Age at diagnosis, years			
Median age (IQR)	65 (56–75)	66 (56–74)	63 (55–73)
20-40	213 (3.45)	13 (3.49)	1 (1.15)
41-60	1,982 (32.14)	118 (31.64)	32 (36.78)
61-80	3,188 (51.70)	197 (52.82)	42 (48.28)
>80	783 (12.70)	45 (12.06)	12 (13.79)
Stage^b^			
Localized	1,848 (29.97)	128 (34.32)	28 (32.18)
Regional	1,931 (31.32)	97 (26.01)	16 (18.39)
Distant	1,920 (31.14)	111 (29.76)	33 (37.93)
Unknown/missing	467 (7.57)	37 (9.92)	10 (11.49)
Comorbidity level^c^			
Low	4,127 (66.93)	251 (67.29)	58 (66.67)
Moderate	1,512 (24.52)	88 (23.59)	26 (29.89)
High	527 (8.55)	34 (9.12)	3 (3.45)
Comorbidities			
Hypertension	681 (11.04)	45 (12.06)	12 (13.79)
Obesity	71 (1.15)	2 (0.54)	0 (0.00)
Atrial fibrillation/flutter	222 (3.60)	22 (5.90)	8 (9.20)
Angina pectoris	257 (4.17)	9 (2.41)	0 (0.00)
Myocardial infarction	160 (2.59)	10 (2.68)	2 (2.30)
Congestive heart failure	183 (2.97)	12 (3.22)	4 (4.60)
Esophageal varices	5 (0.08)	1 (0.27)	0 (0.00)
Tremor	4 (0.06)	0 (0.00)	0 (0.00)
Anxiety	41 (0.66)	1 (0.27)	1 (1.15)
Thyrotoxicosis	182 (2.95)	16 (4.29)	5 (5.75)
COPD	227 (3.68)	13 (3.49)	4 (4.60)
Migraines	78 (1.27)	3 (0.80)	2 (2.30)
Stroke	223 (3.62)	21 (5.63)	4 (4.60)
Chronic kidney disease	81 (1.31)	5 (1.34)	3 (3.45)
Hysterectomy	358 (5.81)	24 (6.43)	7 (8.05)
Tubal sterilization	204 (3.31)	11 (2.95)	3 (3.45)
Comedication use			
ACE-inhibitors	351 (5.69)	104 (27.88)	30 (34.48)
ARBs	198 (3.21)	60 (16.09)	16 (18.39)
Calcium channel blockers	407 (6.60)	112 (30.03)	31 (35.63)
Diuretics	1,276 (20.69)	234 (62.73)	67 (77.01)
Non-aspirin NSAIDs	1,852 (30.04)	220 (58.98)	51 (58.62)
Aspirin	525 (8.51)	157 (42.09)	40 (45.98)
Statins	320 (5.19)	111 (29.76)	28 (32.18)
HRT	1,165 (18.89)	126 (33.78)	33 (37.93)
Antipsychotics	336 (5.45)	41 (10.99)	8 (9.20)
Anxiolytics	285 (4.62)	22 (5.90)	7 (8.05)
Antidepressants	816 (13.23)	100 (26.81)	24 (27.59)

*ACE*: angiotensin-converting enzyme; *ARBs*: angiotensin receptor blockers; *NSAIDs*: nonsteroidal anti-inflammatory drugs.

^a^ Defined as time between first and last prescription plus 90 days (assumed to be the average length of prescription).

^b^ Classified according to Summary Staging classification with the TNM grouping translated as localized (TNM: T1–4, N0, M0), regional (TNM: T1–4, N1–3, M0), distant (TNM: T1–4, N1–3, M1), or unknown/missing.

^c^ Computed using the Charlson Comorbidity Index (CCI) score categorized into low (0), medium (1–2), or high (3+).

 Current and previous users of ß-blockers had more frequently localized stage at the time of diagnosis than nonusers. Stage was unknown/missing for 7.6% of nonusers, 9.9% of current users, and 11.5% of previous users. Nonusers and current users of ß-blockers had similar comorbidity burden whereas previous users had more frequently moderate comorbidity. Current and previous users of ß-blockers more frequently used the comedications identified.

### Mortality

Overall the median follow-up time was 2.55 years (IQR: 0.81-9.23). Compared with nonusers, the adjusted HR was 1.17 (95% CI: 1.02–1.34) for current users and 1.18 (95% CI: 0.90–1.55) for previous users (Table 2). When examining the association according to stage at time of diagnosis, we found similar results (Table 2).

**Table 2:** **Mortality hazard ratio**^**a **^**following ovarian cancer diagnosis associated with ß**-**blocker use**, **overall and by cancer stage at diagnosis**^**b**^

	**Number of deaths(%)**	**Median years of follow**-**up**	**Crude HR** (**95**% **CI**)	**Adjusted HR** (**95**% **CI**)^**c**^
**Overall**				
Nonusers	4,106 (66.59)	2.56	1 (reference)	1 (reference)
Current users	245 (65.68)	2.65	1.04 (0.91–1.18)	1.17 (1.02–1.34)
Previous users	55 (63.22)	2.14	1.03 (0.79–1.35)	1.18 (0.90–1.55)
**Localized cancer**				
Nonusers	722 (39.07)	10.58	1 (reference)	1 (reference)
Current users	46 (35.94)	7.87	0.97 (0.72–1.31)	1.01 (0.73–1.39)
Previous users	13 (46.43)	7.07	1.44 (0.83–2.50)	1.57 (0.90–2.76)
**Regional metastasis**				
Nonusers	1,409 (72.97)	2.54	1 (reference)	1 (reference)
Current users	75 (77.32)	2.21	1.21 (0.96–1.52)	1.52 (1.18–1.95)
Previous users	10 (62.50)	1.42	0.95 (0.51–1.77)	0.89 (0.46–1.69)
**Distant metastasis**				
Nonusers	1,602 (83.44)	1.25	1 (reference)	1 (reference)
Current users	96 (86.49)	1.10	1.09 (0.89–1.35)	1.18 (0.95–1.47)
Previous users	24 (72.73)	1.86	0.70 (0.47–1.05)	0.91 (0.60–1.37)

*CI*: Confidence interval.

^a^ Obtained using Cox proportional hazards models.

^b^ Classified according to Summary Staging classification with the TNM grouping translated as localized (TNM: T1–4, N0, M0), regional (TNM: T1–4, N1–3, M0), distant (TNM: T1–4, N1–3, M1), or unknown/missing.

^c^ Adjusted for age (20–40, 41–60, 61–80, ≥80 years), comorbidity level, prior use of diuretics (yes/no), year of diagnosis, aspirin (yes/no), and statins (yes/no). Comorbidity was computed using the Charlson Comorbidity Index score categorized into low (0), medium (1–2), or high (3+).

There were no statistically significant interactions between any of the covariates and ß-blocker use, except for age. To examine the clinical relevance, we therefore stratified the results by age group. Although rather imprecise, the estimates indicated a tendency towards higher HRs in older age groups (Table 1 in Additional file 2: Tables for secondary analyses and stratification by age). The imprecise estimates for the age group below 40 years, made this strata inconclusive.

The secondary analysis showed that the results for current users with ≥1 year duration of use were not substantially different from the results including all current users (Table 2 in Additional file 2: Additional tables). There was no association between months of use entered as a linear variable and mortality following ovarian cancer (HR 1.01; 95% CI: 1.00–1.01, Table 3 in Additional file 2: Additional tables).

## Discussion

In this population-based cohort study, we found no evidence of an association between ß-blocker use and decreased mortality following a diagnosis of ovarian cancer.

 Previous in vitro data on cancer cell lines suggest that ß-blockers exert an antitumor effect via direct action on ovarian cancer cells [2-4]. It has been shown that treatment with adrenergic agonists could upregulate the production of matrix metalloproteinase (MMP)-2, MMP-9, and VEGF in ovarian cancer cell lines resulting in increased invasive capability [2,4]. This effect was mediated through ß-adrenoceptors and was blocked by treatment with the ß-antagonist propranolol [2,4]. Similar results have been found for other malignancies, *e*.*g*., malignant melanoma, multiple myeloma, and nasopharyngeal carcinoma [5-8,22-25]. Finally, it has been shown that presurgical ovarian cancer patients with low social stress have lower VEGF levels possibly linking stress, and hence ß-adrenergic agents, to tumor angiogenesis [3].

In a recent meta-analysis, the association between ß-blocker use and cancer-related mortality was examined and no association was found (odds ratio=0.93; 95% CI: 0.80–1.08) [26]. However, this study examined only the association for cancer-related mortality overall [26]. To our knowledge, only one previous study has examined this association for ovarian cancer specifically [10]. Diaz *et al*. [10] examined the association between ß-blockers and disease progression and survival among 248 patients with stage III or IV epithelial ovarian or primary peritoneal cancer who had undergone primary exploratory laparotomy followed by at least 6 cycles of platinum- and taxane-based chemotherapy. They found an median survival of 56 months for ß-blocker users compared with 48 months for non-users [10]. However, the interpretabilty of the results is hampered for several reasons. First, the study included a selected patient population, which limits generalizability. Second, the improved survival seemed confined to the later part of follow-up (more than 2–3 years), which is associated with statistical uncertainty because only few patients survived that long. Finally, and most importantly, because authors’ decided that to be considered as exposed a patient had to have used ß-blockers for at least 6 months, they also had to have survived at least 6 months, and therefore immortal time bias may entirely explain the increased survival among ß-blocker users [10,15]. By assessing exposure prior to start of follow-up, we circumvented this problem in our study, which did not support any effect on the mortality rate after ovarian cancer diagnosis associated with prior ß-blocker use. However, we cannot rule out an increased or decreased mortality for individual agents or certain histological types of ovarian cancer. Furthermore, given the wide confidence intervals and the non-randomized design, we are unable to exclude a small protective effect on mortality following ovarian cancer diagnosis.

 Several issues should be considered when interpreting our results. The study’s population-based design within the setting of a tax supported universal healthcare system reduces selection biases. Also, we were able to link population-based registries with complete data on drug use, cancer diagnosis, outpatient visits, and hospitalizations [12,14,19,27,28]. For example, the positive predictive values of diagnoses in the Danish National Registry of Patients have previously been validated and found to exceed 98% for Charlson comorbidities overall [19].

 Data in the prescription database are virtually complete [12]. Although we had to use prescription data as a proxy for actual ß-blocker use, we did not base drug exposure information on prescription filling, but on actual dispensing at pharmacies [12]. Furthermore, we believe that copayment requirements increased the likelihood of compliance. However, any misclassification of drug exposure would most likely have been nondifferential and thus could, in part, explain the null result.

We were only able to consider all-cause mortality. We did not have any biological explanation to support that ß-blocker use would increase mortality rates following ovarian cancer. Thus, multiple comparisons may explain the slightly increased point estimates observed in some exposure categories. These estimates may also have resulted from uncontrolled confounding, as it is plausible that ß-blocker users on average are unhealthier than nonusers. However, the universal provision of health care considerably reduces the likelihood of substantial confounding by social characteristics. Furthermore, we did adjust partly for lifestyle factors by taking a history of obesity, COPD and ischemic heart disease into account. Still, due to the nonrandomized design, we cannot exclude uncontrolled confounding.

## Conclusions

In conclusion, the present population-based cohort study does not provide evidence of an association between ß-blocker use and decreased mortality following a diagnosis of ovarian cancer.

## Competing interests

The authors declare that they have no competing interest.

## Authors’ contributions

RG, EVY, GP, and SL conceived the study idea. All authors reviewed the literature and/or designed the study. SAJ, MS, GP, and SL analyzed the data. All authors interpreted the findings. SAJ and MS organized the writing and wrote the initial draft. All authors edited the manuscript and approved the final version.

## Pre-publication history

The pre-publication history for this paper can be accessed here:

http://www.biomedcentral.com/1471-2407/13/85/prepub

## Supplementary Material

Additional file 1 **ATC and ICD codes. **A table presenting all ATC and ICD codes used in the study. Click here for file

Additional file 2 **Additional tables. **Three tables presenting the results for the secondary analyses and stratification by age. Click here for file
