# Supplementary material for: Use of ß-blockers and mortality following ovarian cancer diagnosis: a population-based cohort study
Source: BMC Cancer. 2013 Feb 22;13:85. doi: 10.1186/1471-2407-13-85 (PMC3598679; doi:10.1186/1471-2407-13-85)
Supplement: Additional file 1 — ATC and ICD codes. A table presenting all ATC and ICD codes used in the study. [file 1471-2407-13-85-S1.docx]

**ADDITIONAL FILE 1 - ATC and ICD codes**

| **Ovarian cancer** | ICD-10: C56 |
| --- | --- |
| **Hypertension (codes specific for hypertension)** | ICD-8: 400-404; ICD-10: I10-I15 |
| **Beta-blockers** | ATC: C07AA01- C07AA07, C07AG01- C07AG02, C07AB02- C07AB12 |
| **Comedication use** |  |
| ACE inhibitors | ATC: C09AA |
| ARBs | ATC: C09CA |
| CCB | ATC: C08C, C08D (except C08DA51) |
| Diuretics | ATC: C03 |
| NSAIDs | ATC: L01XX33, M01AA-M01AH, M01AX01 |
| Statins | ATC: C10AA |
| Hormone replacement therapy | ATC: G03C, G03F |
| Aspirin | ATC: B01AC06, N02BA01, N02BA51 |
| Antipsychotics | ATC: N05A |
| Anxiolytic drugs | ATC: N05B |
| Antidepressant drugs | ATC: N06A |
| Oral contraceptives | ATC: G03A |
| **Charlson Comorbidity Index** |  |
| Score 1 |  |
| Myocardial infarction | ICD-8: 410; ICD-10: I21, I22, I23 |
| Congestive heart failure | ICD-8: 427.09, 427.10, 427.11, 427.19, 428.99, 782.49; ICD-10: I50, I11.0, I13.0, I13.2 |
| Peripheral vascular disease | ICD-8: 440, 441, 442, 443, 444, 445; ICD-10: I70, I71, I72, I73, I74, I77 |
| Cerebrovascular disease | ICD-8: 430-438; ICD-10: I60-I69, G45, G46 |
| Dementia | ICD-8: 290.09-290.19, 293.09; ICD-10: F00-F03, F05.1, G30 |
| Chronic pulmonary disease | ICD-8: 490-493, 515-518, ICD-10: J40-J47, J60-J67, J68.4, J70.1, J70.3, J84.1, J92.0, J96.1, J98.2, J98.3 |
| Connective tissue disease | ICD-8: 712, 716, 734, 446, 135.99; ICD-10: M05, M06, M08, M09, M30, M31, M32, M33, M34, M35, M36, D86 |
| Ulcer disease | ICD-8: 530.91, 530.98, 531-534; ICD-10: K22.1, K25-K28 |
| Mild liver disease | ICD-8: 571, 573.01, 573.04; ICD-10: B18, K70.0-K70.3, K70.9, K71, K73, K74, K76.0 |
| Diabetes type 1 and 2 | ICD-8: 249.00, 249.06, 249.07, 249.09, 250.00, 250.06, 250.07, 250.09; ICD-10: E10.0, E10.1, E10.9, E11.0, E11.1, E11.9 |
| Score 2 |  |
| Hemiplegia | ICD-8: 344; ICD-10: G81, G82 |
| Moderate to severe renal disease | ICD-8: 403, 404, 580-583, 584, 590.09, 593.19, 753.10-753.19, 792; ICD-10: 12, I13, N00-N05, N07, N11, N14, N17-N19, Q61 |
| Diabetes with end organ damage | ICD-8: 249.01-249.05, 249.08, 250.01-250.05, 250.08; ICD-10: E10.2-E10.8, E11.2-E11.8 |
| Any tumor | ICD-8: 140-194 excl. 183.0 and 183.9; ICD-10: C00-C75 excl. C56 |
| Leukemia | ICD-8: 204-207; ICD-10: C91-C95 |
| Lymphoma | ICD-8: 200-203, 275.59; ICD-10: C81-C85, C88, C90, C96 |
| Score 3 |  |
| Moderate to severe liver disease | ICD-8: 070.00, 070.02, 070.04, 070.06, 070.08, 573.00, 456.00-456.09; ICD-10: B15.0, B16.0, B16.2, B19.0, K70.4, K72, K76.6, I85 |
| Score 6 |  |
| Metastatic solid tumor | ICD-8: 195-198, 199; ICD-10: C76-C80 |
| AIDS | ICD-8: 079.83; ICD-10: B21-B24 |
| **Other comorbidities** |  |
| Obesity | ICD-8: 277; ICD-10: E65 |
| Atrial fibrillation/flutter | ICD-8: 427.93-427.94; ICD-10: I48 |
| Angina pectoris | ICD-8: 413; ICD-10: I20 |
| Myocardial infarction | ICD-8: 410; ICD-10: I21-I23 |
| Congestive heart failure | ICD-8: 427.09, 427.10; 427.11, 427.19, 428.99 782.49; ICD-10: I11.0, I13.0, I13.2, I50 |
| Esophageal varices | ICD-8: 456.00, 456.01, 456.09; ICD-10: I85 |
| Tremor | ICD-8: 780.32; ICD-10: G25.0, G25.2, R25.1 |
| Anxiety | ICD-8: 300.09; ICD-10: F40-F41 |
| Thyrotoxicosis | ICD-8: 242; ICD-10: E05, E06.2 |
| COPD | ICD-8: 491-492; ICD-10: J41−J44 |
| Migraines | ICD-8: 346; ICD-10: G43, G43.0, G43.0A, G43.1, G43.1A, G43.1B, G43.1C, G43.2, G43.3, G43.8, G43.9 |
| Stroke | ICD-8: 431, 433-434; ICD-10: I61, I63-I64 |
| Chronic kidney disease | ICD-8: 249.02, 250.02, 753.10-753.19, 582, 583, 584, 590.09, 593.20, 792; ICD-10: E10.2, E11.2, E14.2, N03, N05, N11.0, N14; N16, N18-N19, N26.9, Q61.1-Q61.4 |
| Hysterectomy | Danish Classification of Surgical Procedures: 610.00, 610.20, 610.40; NOMESCO Classification of Surgical Procedures: KLCD |
| Tubal sterilization | Danish Classification of Surgical Procedures: 608.00, 608.10, 608.20, 608.30, 608.40; NOMESCO Classification of Surgical Procedures: KLG |
