# Supplementary material for: Use of ß-blockers and mortality following ovarian cancer diagnosis: a population-based cohort study
Source: BMC Cancer. 2013 Feb 22;13:85. doi: 10.1186/1471-2407-13-85 (PMC3598679; doi:10.1186/1471-2407-13-85)
Supplement: Additional file 2 — Additional tables. Three tables presenting the results for the secondary analyses and stratification by age. [file 1471-2407-13-85-S2.docx]

**ADDITIONAL FILE 2 – Additional tables**

## Table 1 – Adjusted mortality hazard ratio (HR)^a^ following ovarian cancer diagnosis associated with ß-blocker use, by age at diagnosis

|  | **Number of deaths (%)** | **Median years of follow-up** | **Crude HR (95% CI)** | **Adjusted HR (95% CI)**^b^ |
| --- | --- | --- | --- | --- |
| **20-40 years** |  |  |  |  |
| Nonusers | 51 (23.94) | 4.80 | 1 (reference) | 1 (reference) |
| Current users | 5 (38.46) | 5.68 | 2.21 (0.88–5.57) | 2.03 (0.71–5.84) |
| Previous users | – | 7.07 | – | – |
| **41-60 years** |  |  |  |  |
| Nonusers | 1,054 (53.18) | 4.80 | 1 (reference) | 1 (reference) |
| Current users | 60 (50.85) | 5.68 | 0.98 (0.75–1.27) | 1.03 (0.78–1.37) |
| Previous users | 16 (50.00) | 7.07 | 1.06 (0.65–1.74) | 1.18 (0.71–1.95) |
| **61-80 years** |  |  |  |  |
| Nonusers | 2,300 (72.15) | 2.12 | 1 (reference) | 1 (reference) |
| Current users | 138 (70.05) | 1.86 | 1.03 (0.87–1.22) | 1.14 (0.95–1.37) |
| Previous users | 28 (66.67) | 1.78 | 0.98 (0.68–1.42) | 1.10 (0.75–1.62) |
| **>80 years** |  |  |  |  |
| Nonusers | 701 (89.53) | 0.54 | 1 (reference) | 1 (reference) |
| Current users | 42 (93.33) | 0.30 | 1.25 (0.91–1.71) | 1.50 (1.07–2.11) |
| Previous users | 11 (91.67) | 0.21 | 1.29 (0.71–2.33) | 1.52 (0.81–2.83) |
| CI: Confidence interval  ^a^ Obtained using Cox proportional hazards models  ^b^ Adjusted for comorbidity level, prior use of diuretics (yes/no), year of diagnosis, aspirin (yes/no), and statins (yes/no). Comorbidity was computed using the Charlson Comorbidity Index score categorized into low (0), medium (1-2), or high (3+). | | | | |

## Table 2 – Adjusted hazard ratio (HR)^a^ following ovarian cancer diagnosis associated with continuing ß-blocker use among current users, overall and by cancer stage at diagnosis^b^

|  | **Number of deaths (%)** | **Median years of follow-up** | **Crude HR (95% CI)** | **Adjusted HR (95% CI)^c^** |
| --- | --- | --- | --- | --- |
| **Overall** |  |  |  |  |
| Nonusers | 4,106 (66.59) | 2.56 | 1 (reference) | 1 (reference) |
| Current users with ≥1 years duration of use | 149 (68.35) | 2.60 | 1.10 (0.93–1.29) | 1.25 (1.05–1.49) |
| **Localized cancer** |  |  |  |  |
| Nonusers | 722 (39.07) | 10.58 | 1 (reference) | 1 (reference) |
| Current users with ≥1 years duration of use | 27 (37.50) | 7.31 | 1.03 (0.70–1.51) | 1.06 (0.70–1.59) |
| **Regional metastasis** |  |  |  |  |
| Nonusers | 1,409 (72.97) | 2.54 | 1 (reference) | 1 (reference) |
| Current users with ≥1 years duration of use | 42 (77.78) | 2.65 | 1.16 (0.86–1.58) | 1.62 (1.16–2.25) |
| **Distant metastasis** |  |  |  |  |
| Nonusers | 1,602 (83.44) | 1.25 | 1 (reference) | 1 (reference) |
| Current users with ≥1 years duration of use | 65 (90.28) | 1.10 | 1.12 (0.87–1.43) | 1.14 (0.88–1.49) |
| CI: Confidence interval  ^a^ Obtained using Cox proportional hazards models  ^b^ Classified according to Summary Staging classification with the TNM grouping translated as localized (TNM: T1–4, N0, M0), regional (TNM: T1–4, N1–3, M0), distant (TNM: T1–4, N1–3, M1), or unknown/missing.  ^c^ Adjusted for age (20-40, 41-60, 61-80, ≥80 years), comorbidity level, prior use of diuretics (yes/no), year of diagnosis, aspirin (yes/no), and statins (yes/no). Comorbidity was computed using the Charlson Comorbidity Index score categorized into low (0), medium (1-2), or high (3+). | | | | |

## Table 3 – Adjusted hazard ratio (HR)^a^ following ovarian cancer diagnosis associated with duration of ß-blocker use, overall and by cancer stage at diagnosis^b^

|  | **Crude HR (95% CI)** | **Adjusted HR (95% CI)^c^** |
| --- | --- | --- |
| **Overall** |  |  |
| Nonusers | 1 (reference) | 1 (reference) |
| Months of use | 1.00 (1.00–1.00) | 1.00 (1.00–1.01) |
| **Localized cancer** |  |  |
| Nonusers | 1 (reference) | 1 (reference) |
| Months of use | 1.00 (1.00–1.01) | 1.01 (1.00–1.01) |
| **Regional metastasis** |  |  |
| Nonusers | 1 (reference) | 1 (reference) |
| Months of use | 1.00 (1.00–1.01) | 1.00 (1.00–1.01) |
| **Distant metastasis** |  |  |
| Nonusers | 1 (reference) | 1 (reference) |
| Months of use | 1.00 (1.00–1.00) | 1.00 (1.00–1.01) |
| CI: Confidence interval  ^a^ Obtained using Cox proportional hazards models  ^b^ Classified according to Summary Staging classification with the TNM grouping translated as localized (TNM: T1–4, N0, M0), regional (TNM: T1–4, N1–3, M0), distant (TNM: T1–4, N1–3, M1), or unknown/missing.  ^c^ Adjusted for age (20-40, 41-60, 61-80, ≥80 years), comorbidity level, prior use of diuretics (yes/no), year of diagnosis, aspirin (yes/no), and statins (yes/no). Comorbidity was computed using the Charlson Comorbidity Index score categorized into low (0), medium (1-2), or high (3+). | | |
